# Outcomes of extracorporeal membrane oxygenation following the 2018 adult heart allocation policy

**DOI:** 10.1371/journal.pone.0268771

**Published:** 2022-05-20

**Authors:** Samuel T. Kim, Yu Xia, Zachary Tran, Joseph Hadaya, Vishal Dobaria, Chun Woo Choi, Peyman Benharash

**Affiliations:** 1 Division of Cardiac Surgery, Cardiovascular Outcomes Research Laboratories (CORELAB), David Geffen School of Medicine at UCLA, Los Angeles, CA, United States of America; 2 Division of Cardiovascular Surgery, Johns Hopkins University School of Medicine, Baltimore, MD, United States of America; National Taiwan University Hospital, TAIWAN

## Abstract

**Background:**

The purpose of the study was to characterize changes in waitlist and post-transplant outcomes of extracorporeal membrane oxygenation (ECMO) patients bridged to heart transplantation under the 2018 adult heart allocation policy.

**Methods:**

All adult patients listed for isolated heart transplantation from August 2016 to December 2020 were identified using the United Network for Organ Sharing database. Patients were stratified into Eras (Era 1 and Era 2) centered around the policy change on October 18, 2018. Competing risk regression was used to evaluate waitlist death or deterioration across Eras. Cox proportional hazards models were used to determine associations between use of ECMO and 1-year post-transplant mortality within each Era.

**Results:**

Of 8,902 heart transplants included in analysis, 339 (3.8%) were bridged with ECMO (Era 2: 6.1% vs Era 1: 1.2%, P<0.001). Patients bridged with ECMO in Era 2 were less frequently female (26.0% vs 42.0%, P = 0.02) and experienced shorter waitlist times (5 vs 11 days, P<0.001) along with a lower likelihood of waitlist death or deterioration (subdistribution hazard ratio, 0.45, 95% confidence interval, CI, 0.30–0.68, P<0.001) compared to those in Era 1. Use of ECMO was associated with increased post-transplant mortality at 1-year compared to all other transplants in Era 1 (hazard ratio 3.78, 95% CI 1.88–7.61, P < 0.001) but not Era 2.

**Conclusions:**

Patients bridged with ECMO in Era 2 experience improved waitlist and post-transplant outcomes compared to Era 1, giving credence to the increased use of ECMO under the new allocation policy.

## Introduction

The Organ Procurement and Transplantation Network (OPTN) implemented drastic changes to the existing adult heart allocation policy on October 18, 2018 [1, 2]. Prior to this change, heart transplant candidates were categorized into three broadly-defined tiers, with the majority falling into the highest priority status [1]. The new policy was aimed at prioritizing the most urgent patients with emphasis on the duration and type of mechanical circulatory support (MCS) being used. Under the new policy, stable and dischargeable patients with left ventricular assist devices (LVADs) received a decrement in urgency, while those on extracorporeal membrane oxygenation (ECMO) and intra-aortic balloon pumps (IABP) were prioritized in status.

Following these changes, several groups have reported a significant decrease in the nationwide use of LVADs on the waitlist [3, 4]. Conversely, temporary MCS (tMCS) devices such as ECMO and IABP are increasingly used as bridge to transplantation [3, 4]. While such changes in MCS use under the new system have been previously described [4–8], little is known regarding the characteristics and outcomes of the large influx of patients now being bridged with ECMO in the new allocation scheme. The present study characterized changes in waitlist and post-transplant outcomes of ECMO patients being bridged to transplantation following the 2018 OPTN policy change. We hypothesized that under this new policy, ECMO patients would experience improved survival both on the waitlist and following transplantation due to prioritization.

## Methods

### Study population

This was a retrospective cohort study using the United Network for Organ Sharing (UNOS) database from August 2016 to December 2020. All adults (≥18 years) listed for isolated heart transplantation were stratified into equal time cohorts with Era 1 denoting the period prior to October 18, 2018 and Era 2 for the period after. For analysis of waitlist outcomes, eras were determined based on the date of listing, with Era 1 patients being censored on October 17, 2018. Similarly, date of transplantation was used to stratify patients for analysis of post-transplant outcomes, while excluding subjects listed in Era 1 but transplanted in Era 2. The primary endpoint of the study was survival at one year following transplantation while secondary outcomes included 30-day mortality following transplantation, waitlist death or deterioration and waitlist duration.

### Statistical analysis

Differences between Eras were compared using the Mann-Whitney-U and Chi-square tests for continuous and categorical variables, respectively. Continuous variables are reported as medians with interquartile ranges (IQR), while categorical variables are shown as counts with proportions of the cohort. The significance of post-transplant survival across cohorts was assessed using the log-rank test and visualized using Kaplan-Meier survival estimates.

Multivariable Cox proportional-hazards models were used to evaluate the association of the policy change on adjusted post-transplant mortality. Schoenfeld residuals were visualized for each covariate to assess the validity of the proportional-hazards assumption, with all covariates meeting the requirement. Using multivariable logistic regression, we additionally evaluated associations between patient-level factors and adjusted probability of post-transplant mortality at 30-days. Covariates for Cox proportional-hazards and logistic regression models are reported as hazard ratios (HR) and odds ratios (OR), respectively. To assess differences in waitlist death or clinical deterioration by Era, competing risk subdistribution hazard (SHR) models were constructed using Fine-Gray competing-risks regressions [9].

Covariates for multivariable models were selected using the Elastic net regularization method. Briefly, this method selects covariates and accounts for collinearity by using a penalized least squares mode of selection [10]. Final variables adjusted for included recipient age, gender, body mass index (BMI), dialysis status, ventilator use at transplant, cerebrovascular disease, prior cardiac surgery, Karnofsky functional status, serum creatinine, serum total bilirubin, systolic pulmonary artery (PA) pressure and cardiac output. All statistical analyses were performed using Stata 16 (StataCorp LLC, College Station, TX) with an α-level of < 0.05 considered significant for all tests.

The study was deemed exempt from full review by the Institutional Review Board at the University of California, Los Angeles and informed consent was not acquired due to the publicly available, deidentified nature of the dataset. This in full accordance with the United States Health and Human Services regulations, 45 CFR Part 46.

## Results

### Baseline characteristics of ECMO patients bridged to heart transplant across eras

Of 15,897 patients considered for analysis, 8,902 (56.0%) received a heart transplant. A higher proportion of heart transplants were bridged with ECMO in Era 2 compared to Era 1 (Era 2: 6.1% vs Era 1: 1.2%, P < 0.001) (Table 1). Among those bridged with ECMO, Era 2 recipients were less commonly female (26.0% vs 42.0%, P = 0.02) compared to Era 1. Era 2 donors were also less frequently female (19.7% vs 46.0%, P < 0.001), had significantly longer cold ischemic times (3.4 (2.8–3.9) hours vs 2.8 (IQR: 2.2–3.6) hours, P < 0.001) and were transported longer distances (256 (102–424) miles vs 63.5 (7–293) miles, P < 0.001) compared to donors in Era 1. Rates of dialysis, intra-aortic balloon pump and ventilator use among recipients remained similar across cohorts, as did the presence of comorbidities such as diabetes, pulmonary hypertension and cerebrovascular disease (Table 1). Similar findings were observed across Eras among patients on ECMO at listing, as shown in S1 Table.

**Table 1:** Baseline characteristics of patients on ECMO at transplant.

Variable	Era 1	Era 2	p-value
	n = 50	n = 289	
Proportion of all Heart Recipients	1.2%	6.1%	< 0.001
*Recipient Characteristics*			
Age, y	49 (31–61)	50 (34–60)	0.67
Female	42.0%	26.0%	0.02
Non-White race	22.0%	35.6%	0.06
BMI	27.3 (23.0–32.8)	26.7 (23.7–30.5)	0.50
Days on waitlist, d	11 (5–39)	5 (2–13)	< 0.01
Ventilator use at transplant	30.0%	30.8%	0.91
IABP at transplant	14.0%	21.1%	0.31
Inotropes at transplant	52.0%	52.9%	0.90
Dialysis while listed	14.0%	11.8%	0.65
Diabetes	14.0%	21.1%	0.25
Pulmonary Hypertension*	72.0%	73.7%	0.80
Cerebrovascular Disease	8.0%	4.5%	0.30
Prior Cardiac Surgery	32.0%	31.5%	0.94
Functional Status, (1–10)**	2 (1.5–2)	2 (2–2)	0.09
Serum total bilirubin, mg/dL	1.1 (0.7–2.3)	1.1 (0.7–1.9)	0.88
Serum creatinine, mg/dL	1 (0.7–1.5)	1 (0.72–1.5)	0.94
Systolic PA Pressure, mmHg	41.5 (30–49)	41 (31–51.5)	0.54
Mean PA Pressure, mmHg	28.5 (23–37)	30 (23–38)	0.54
Cardiac Output, L/min	3.7 (2.9–4.4)	3.7 (2.9–4.8)	0.60
*Donor Characteristics*			
Female	46.0%	19.7%	< 0.001
Age, y	34 (23–43)	30 (24–37)	0.21
Hypertension	46.0%	36.3%	0.19
Diabetes	4.0%	1.7%	0.30
Ischemic time, h	2.8 (2.2–3.6)	3.4 (2.8–3.9)	< 0.001
Distance, miles	63.5 (7–293)	256 (102–424)	< 0.001

Values are expressed as median ± interquartile range or percentages

BMI = body mass index; ECMO = Extracorporeal Mechanical Oxygenation; IABP = Intraortic balloon pump; PA = Pulmonary Artery

*Pulmonary Hypertension defined as mean PA pressure ≥ 25 mmHg

**Karnofsky functional status; lower numbers denote sicker patients

### Waitlist outcomes

**Fig 1:**
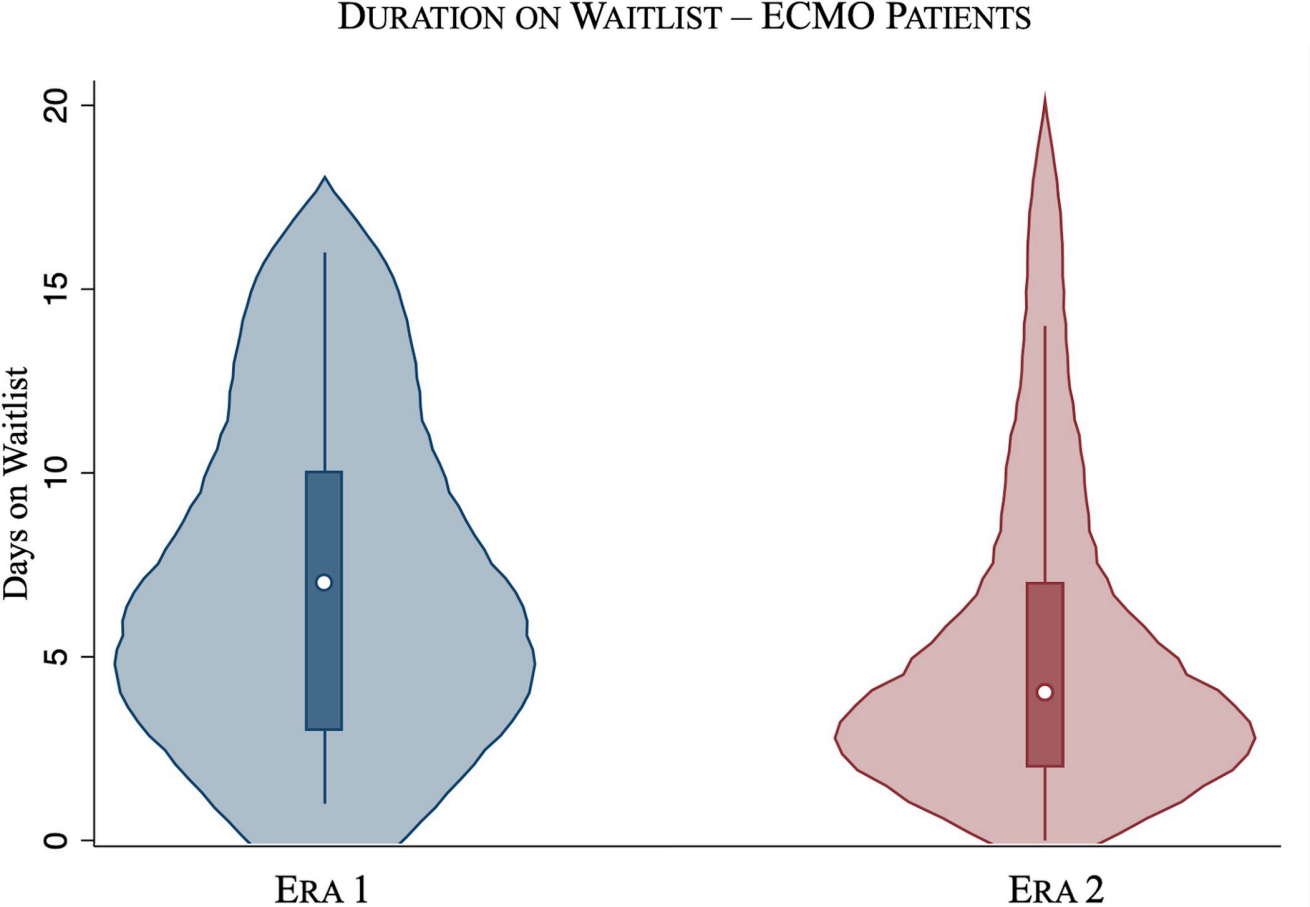
Violin plot of waitlist times for those bridged to transplantation in Era 1 (left) vs Era 2 (right).

**Fig 2:**
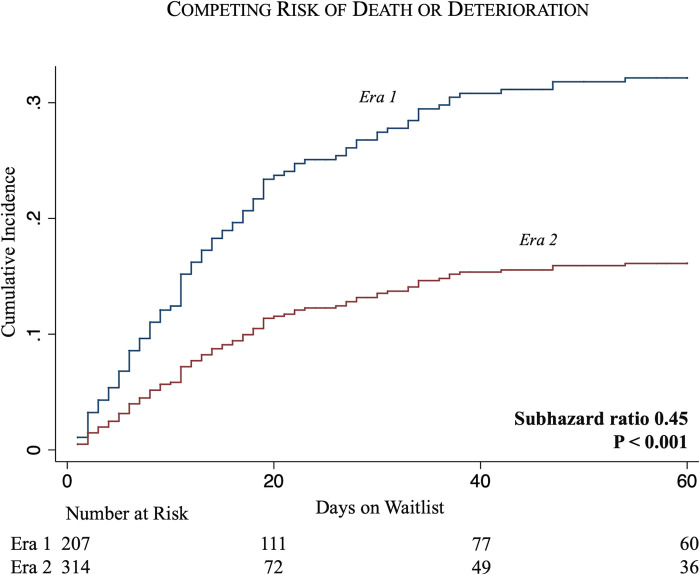
Competing risks regressions for waitlist death or deterioration among all patients listed with ECMO.

Among patients bridged to transplantation with ECMO, Era 2 recipients experienced shorter median waitlist times (5 days vs 11 days, P < 0.001) compared Era 1. Additionally, the proportion of recipients spending ≤3 days on the waitlist increased across Eras (18.0% in Era 1 to 36.3% in Era 2, P < 0.01), with a decrease in the proportion spending >15 days on the waitlist (36.0% in Era 1 to 21.1% in Era 2, P = 0.02) (Fig 1). Bridging with ECMO in Era 2 was associated with a lower likelihood of death or clinical deterioration on the waitlist (SHR 0.45, 95% CI 0.30–0.68, P < 0.001) compared to Era 1 (Fig 2).

### Post-transplant outcomes

**Fig 3:**
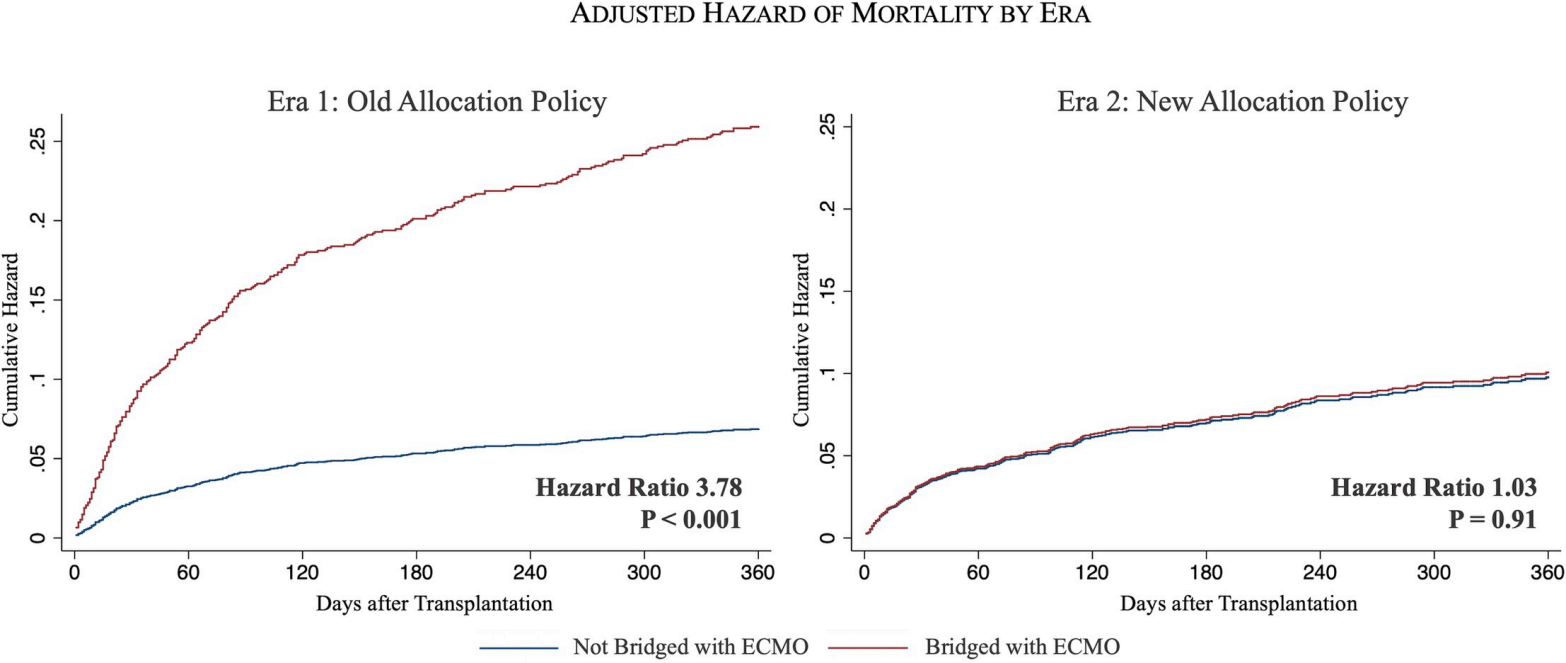
Adjusted hazard of mortality at 1-year following transplantation among those bridged with ECMO (red) vs not bridged with ECMO (blue). Era 1 shown on left panel and Era 2 shown on right.

Bridging with ECMO in Era 1 was associated with an increased hazard of mortality at 1-year following transplantation (HR 3.78, 95% CI 1.88–7.61, P < 0.001) compared to all other heart transplants (S2 Table). However, bridging with ECMO in Era 2 showed a similar hazard of mortality compared to the non-ECMO cohort (HR 1.03, 95% CI 0.60–1.76, P = 0.91) (S3 Table, Fig 3).

**Fig 4:**
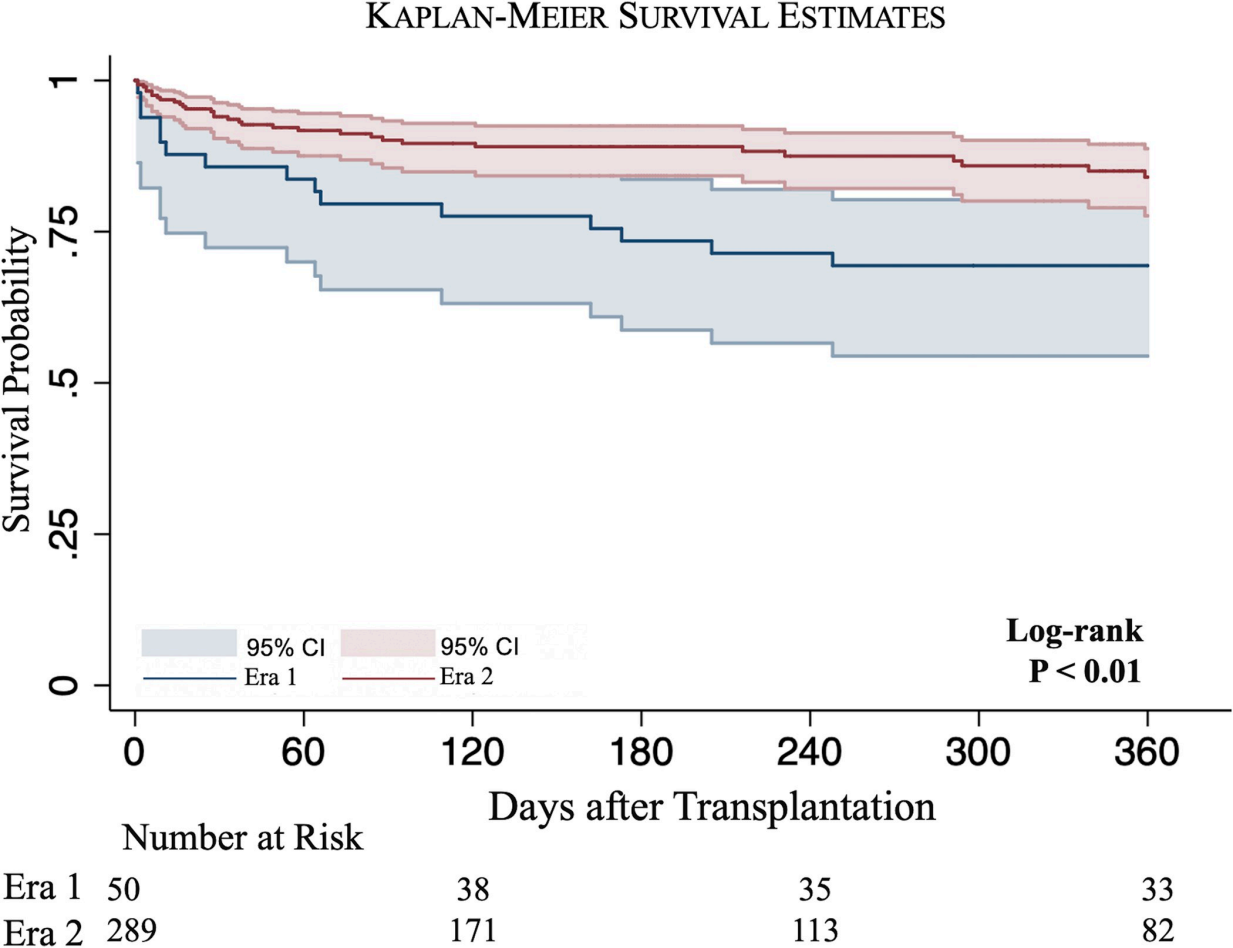
Kaplan-Meier survival estimates of all patients bridged to ECMO in Era 1 (blue) vs Era 2 (red).

**Fig 5:**
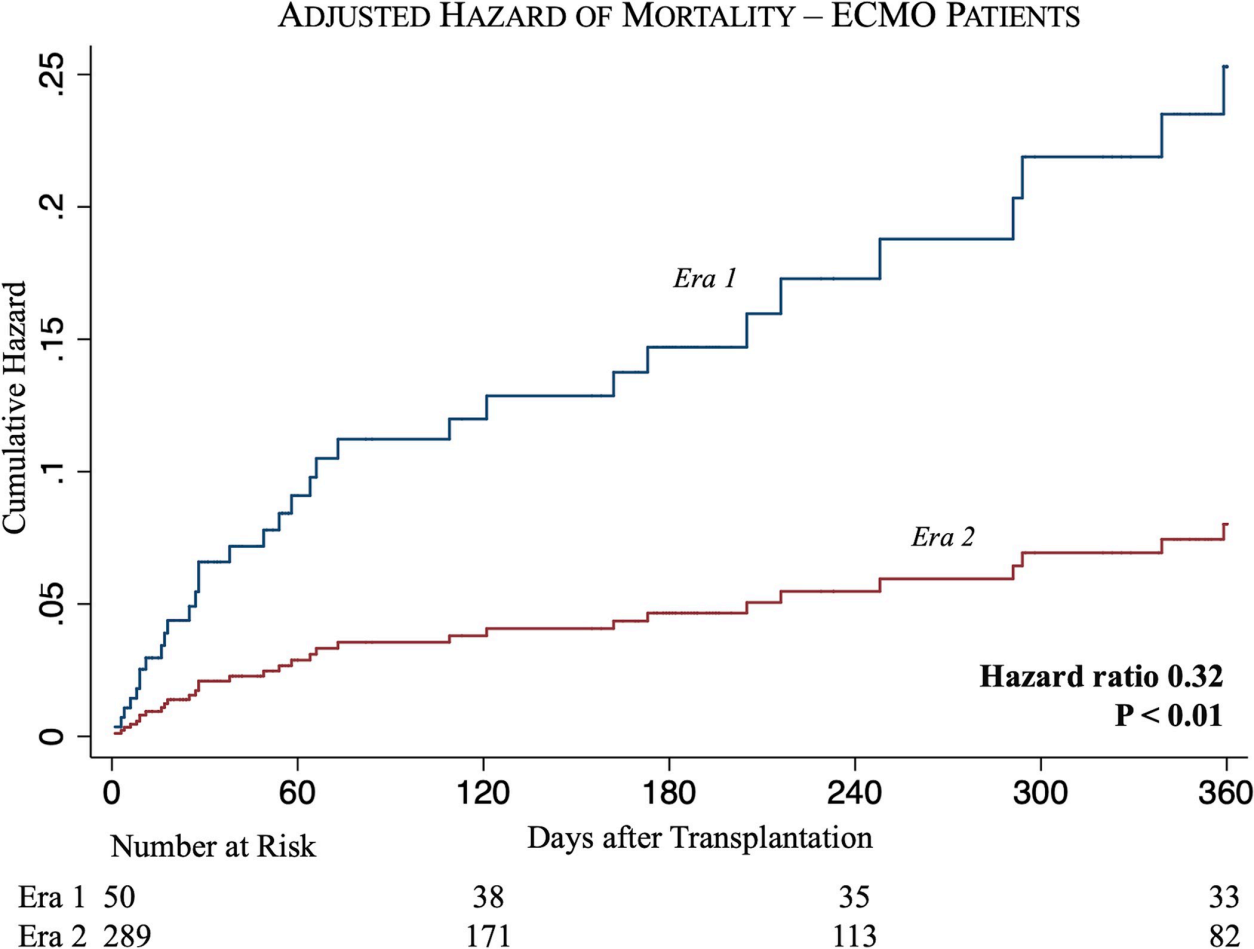
Adjusted cumulative hazard functions for 1-year mortality among all patients bridged to ECMO in Era 1 (blue) vs Era 2 (red).

A subgroup analysis of patients bridged with ECMO revealed lower rates of unadjusted 30-day post-transplant mortality in Era 2 compared to Era 1 (5.9% vs 16.0%, P = 0.01) (Table 2). On Kaplan-Meier analysis, Era 2 recipients experienced increased unadjusted survival over Era 1 recipients at 3 months (90.1% vs 79.6%, P = 0.03), 6 months (89.1% vs 73.5%, P < 0.01) and 1 year (84.0% vs 69.4%, P < 0.01) following transplantation (Fig 4). Adjusted analysis was performed at 1-year following transplantation and showed bridging with ECMO in Era 2 to remain associated with a lower hazard of post-transplant mortality compared to Era 1 (HR 0.32, 95% CI 0.14–0.71, P < 0.01) (S4 Table, Fig 5).

**Table 2:** Survival of ECMO patients bridged to heart transplant.

Variable	Era 1	Era 2	p-value
	n = 50	n = 289	
30-day mortality	16.0%	5.9%	0.01
Graft Failure	2.0%	1.0%	0.56
Retransplantation	0.0%	1.0%	0.47
Postoperative Stroke	8.0%	9.0%	0.82
Postoperative Pacemaker	2.0%	1.0%	0.56
*Survival*			
3-month	79.6%	90.1%	0.03
6-month	73.5%	89.1%	< 0.01
1-year	69.4%	84.0%	< 0.01

### Predictors of 30-day mortality among heart recipients bridged with ECMO

As shown in Table 3, several recipient-level features were independently associated with increased odds of 30-day post-transplant mortality in the ECMO cohort (C-statistic 0.905). Notably, increasing recipient age (OR 1.06/year, 95% CI 1.00–1.12, P = 0.05) and BMI (OR 1.30, 95% CI 1.09–1.54, P < 0.01), as well as ventilator use at transplant (OR 8.45, 95% CI 2.13–33.55, P < 0.01) and prior cardiac surgery (OR 7.03, 95% CI 1.67–29.68, P < 0.01) exhibited independent associations with adjusted 30-day mortality. However, recipient sex, dialysis status, cerebrovascular disease, functional status, serum creatinine, serum total bilirubin, systolic PA pressure and cardiac output were not significantly associated with increased mortality.

**Table 3:** Risk prediction model for 30-day mortality in patients bridged with ECMO.

Recipient Variable	Hazard Ratio	95% CI	p-value
Age, per 1 y	1.06	1.00–1.12	0.05
Female	0.30	0.03–3.05	0.31
Body mass index, per 1 kg/m2	1.30	1.09–1.54	< 0.01
Ventilator use at transplant	8.45	2.13–33.55	< 0.01
Prior cardiac surgery	7.03	1.67–29.68	< 0.01
Dialysis	0.14	0.02–1.38	0.09
Cerebrovascular disease	2.65	0.33–21.5	0.36
Functional status, per 1 u*	1.02	0.71–1.45	0.93
Serum creatinine, per 1 mg/dL	2.37	0.93–6.03	0.07
Serum total bilirubin, per 1 mg/dL	0.92	0.71–1.21	0.56
Systolic PA pressure, per 1 mmHg	1.04	0.99–1.86	0.13
Cardiac output, per 1 L/min	1.32	0.94–1.86	0.11

BMI = body mass index; ECMO = Extracorporeal Mechanical Oxygenation; PA = Pulmonary Artery

*Karnofsky functional status; lower numbers denote sicker patients

## Discussion

As use of tMCS continues to grow under the 2018 adult heart allocation policy, analysis of outcomes is increasingly pertinent in this patient cohort. The present study examined changes in waitlist and post-transplant outcomes of ECMO patients bridged to heart transplantation under the new allocation scheme. We found that ECMO patients in Era 2 experienced shorter waitlist times as well as improved waitlist mortality and post-transplant survival despite similar acuity to Era 1. While use of ECMO as bridge to transplant was associated with worse post-transplant outcomes in Era 1, this association was not apparent in Era 2. Finally, we found several recipient-level factors to be strongly associated with 30-day mortality among patients bridged with ECMO. These findings may aid in candidate selection and prioritization as the role of tMCS in heart transplantation continues to grow.

Our findings expand on work by Kilic and colleagues, who found improved waitlist outcomes but *similar* post-transplant mortality for ECMO patients following the rule change [5]. Our study is the first to report an *improvement* in post-transplant mortality for any cohort under the new policy, with several studies showing the opposite trend for the overall heart transplant population [4, 7, 8]. This difference may reflect the increased follow-up period included in our study compared to those by Kilic et al. Our study period includes data up to December 2020, while investigation by Kilic and colleagues ends in January 2020. Improvements in post-transplant survival may also reflect advances in the pre- and post-transplant management of ECMO patients in recent years. To our knowledge, this is the first study to a) report decreased 1-year post-transplant mortality for ECMO patients in the new era, b) show similar post-transplant mortality between ECMO and non-ECMO transplant recipients following the rule change and c) investigate factors associated with 30-day post-transplant mortality in a contemporary cohort of patients bridged with ECMO. Many have cited the increased proportion being bridged with tMCS to be behind the increase in mortality, as tMCS modalities such as ECMO have classically been associated with poor outcomes following heart transplantation [11, 12]. However, our results show bridging with ECMO to no longer be a predictor of inferior outcomes, as ECMO patients exhibited similar post-transplant mortality as others in the new Era. When taken together, these findings give further credence to the increased use of ECMO as a safe and effective bridging modality under the new scheme.

The improvement in post-transplant mortality in the ECMO cohort may be attributable to several factors, including a decrease in median waitlist time as reported in our study. Longer duration on ECMO has previously been associated with worse mortality and poor outcomes, with recent calls to decrease time to transplant among patients on ECMO [11, 12]. While time on ECMO was not available in the UNOS database, our study found waitlist duration to be reduced by more than half in the current Era. Shorter waitlist duration is likely to correlate with less time on ECMO and may accordingly be a driving factor for our findings. Alternatively, it is also possible that ECMO may be used more liberally in recent years, with some degree of patient selection bias in the new Era contributing to better outcomes. While our findings show ECMO patients under the new policy to have a similar risk profile as the prior Era, it is possible that there were improvements in more granular clinical characteristics not tracked by UNOS. Such characteristics may include differences in echocardiographic abnormalities, kidney function and certain lab values, among others. Finally, improvements in pre- and post-operative management of ECMO patients in recent years may also be playing a role in the improvement in post-transplant survival. Given that ECMO patients have similar post-transplant outcomes to non-bridged recipients in the new era, physicians may consider reducing the use of more complex bridging modalities such as LVAD in the setting of cardiogenic shock.

Given the large influx of patients bridged with ECMO, it is increasingly important to characterize the factors associated with early mortality in this cohort. An informed assessment of mortality risk for patients on ECMO may facilitate appropriate organ allocation for this growing proportion of the waitlist. Previous attempts to characterize predictors of mortality occurred prior to the rule change and may not adequately delineate patients in the modern era [12, 13]. In our study, post-transplant survival was superior in Era 2, despite a significantly longer median organ ischemic time. This may suggest that transplant outcomes are swayed more heavily by recipient risk factors over donor variables–perhaps due to improvements in protecting end-organ perfusion with tMCS and its earlier, more judicious application. Several recipient factors were strongly associated with increased mortality at 30-days following transplantation, including prior cardiac surgery, ventilator status at transplant and morbid obesity. The risk factors for ECMO transplant recipients can be contrasted to those of the general population, which have been previously described [14, 15]. Notably, increased BMI was a strong risk factor of early mortality in ECMO patients bridged to transplant. This contrasts with the total population, in which obesity has no association with worse outcomes or has even been reported to have a protective effect [16–19]. The detriment of increased BMI in the setting of ECMO may be due to problems with vascular access, patient positioning and difficulty attaining adequate perfusion of excess tissue [20]. However, these data should not dissuade use of ECMO in the morbidly obese, as previous data suggests its efficacy as a general form of life support [20–22]. Rather, physicians should exercise caution when deciding to transplant ECMO patients exhibiting any of the significant risk factors in our model.

Several limitations exist in our study, including those that are inherent to a large-scale national database such as UNOS. Notably, we were not able to adjust for granular clinical characteristics including left ventricular hypertrophy, interventricular septum thickness or echocardiographic wall abnormalities which have all been associated with worse outcomes. We were not able to analyze time spent on ECMO or other MCS modalities, as this data was not readily available in the UNOS database. Furthermore, we do not have information on physician-level decision making or center-level policies surrounding MCS use, which may play a large factor when choosing between bridging modalities. This study overlaps with the coronavirus disease 2019 pandemic, which has had a significant impact on heart transplant practices during its early stages [23, 24]. However, recent studies have largely shown a national recovery of heart transplant volume following a nadir early on in the pandemic [23, 24]. Lastly, the final model for 1-year post-transplant mortality contains 13 variables, which may present a risk of overfitting given the smaller sample size of our study. Further investigation should aim to evaluate the impact of the rule change on ECMO with larger cohorts as more data becomes available.

In this retrospective cohort study, we observed improved waitlist and post-transplant outcomes in patients bridged with ECMO following the heart allocation policy change. While bridging with ECMO was strongly associated with worse 1-year mortality prior to the policy change, ECMO patients have similar post-transplant outcomes as non-ECMO patients in the new Era. These findings suggest that ECMO may be most effective as a bridging modality when patients are transplanted in a timely manner following initial listing, though liberalization of ECMO patient selection is also a possible explanation. Further investigation into risk factors associated with early post-transplant mortality among patients on ECMO is warranted, given its growing use as a bridge to heart transplantation.

## Supporting information

S1 Table Baseline characteristics of patients on extracorporeal membrane oxygenation (ECMO) at listing. (DOCX)

S2 Table Cox proportional hazards model of 1-year post-transplant mortality in Era 1 (prior to 2018 adult heart allocation policy change). (DOCX)

S3 Table Cox proportional hazards model of 1-year post-transplant mortality in Era 2 (following 2018 adult heart allocation policy change). (DOCX)

S4 Table Cox proportional hazards model of 1-year post-transplant mortality of extracorporeal membrane oxygenation patients. (DOCX)

## Abbreviations and acronyms

BMI: Body mass index
ECMO: Extracorporeal membrane oxygenation
HR: Hazard ratio
IABP: Intra-aortic balloon pump
IQR: Interquartile range
LVAD: Left ventricular assist device
OPTN: Organ Procurement and Transplantation Network
OR: Odds ratio
PA: Pulmonary artery
SHR: Subdistribution hazard ratio
tMCS: Temporary mechanical circulatory support
UNOS: United Network for Organ Sharing
