# Supplementary material for: Outcomes of extracorporeal membrane oxygenation following the 2018 adult heart allocation policy
Source: PLoS One. 2022 May 20;17(5):e0268771. doi: 10.1371/journal.pone.0268771 (PMC9122227; doi:10.1371/journal.pone.0268771)
Supplement: S1 Table — (DOCX) [file pone.0268771.s001.docx]

| Supplementary Table 1. Baseline Characteristics of Patients on ECMO at Listing | | | |
| --- | --- | --- | --- |
| Variable | Era 1 n = 157 | Era 2 n = 265 | p-value |
| Proportion of all Listings | 2.0% | 3.3% | < 0.001 |
|  |  |  |  |
| *Recipient Characteristics* |  |  |  |
| Age, y | 49 (34 – 59) | 53 (35 - 60) | 0.25 |
| Female | 31.9% | 30.2% | 0.72 |
| Non-White Minority | 29.3% | 33.6% | 0.36 |
| BMI | 27.8 (23.9 - 32.5) | 27.0 (24.2 - 31.5) | 0.36 |
|  |  |  |  |
| Ventilator at listing | 35.0% | 39.6% | 0.35 |
| IABP at listing | 16.6% | 18.1% | 0.69 |
| Inotropes at listing | 42.7% | 54.3% | 0.02 |
| Diabetes | 17.2% | 19.6% | 0.54 |
| Pulmonary Hypertension* | 73.9% | 70.9% | 0.52 |
| Cerebrovascular Disease | 6.4% | 5.7% | 0.77 |
| Prior Cardiac Surgery | 32.5% | 34.0% | 0.76 |
| Functional Status, (1-10)** | 2 (1 - 2) | 2 (1 - 2) | 0.33 |
|  |  |  |  |
| Serum creatinine, mg/dL | 1.1 (0.8 - 1.6) | 1.1 (0.8 - 1.6) | 0.24 |
| Systolic PA Pressure, mmHg | 41 (27 - 50) | 39 (29 - 50) | 0.72 |
| Mean PA Pressure, mmHg | 29 (22 - 37) | 29 (20 - 37) | 0.82 |
| Cardiac Output, L/min | 3.8 (2.7 – 5.2) | 3.8 (2.9 - 5.3) | 0.66 |
| *Values are expressed as median ± interquartile range or percentages* | | | |
| *BMI = body mass index; ECMO = Extracorporeal Mechanical Oxygenation; IABP = Intraaortic balloon pump; PA = Pulmonary Artery* | | | |
| **Pulmonary Hypertension defined as mean PA pressure ≥ 25 mmHg; **Karnofsky functional status; lower numbers denote sicker patients* | | | |
