# Supplementary material for: Outcomes of extracorporeal membrane oxygenation following the 2018 adult heart allocation policy
Source: PLoS One. 2022 May 20;17(5):e0268771. doi: 10.1371/journal.pone.0268771 (PMC9122227; doi:10.1371/journal.pone.0268771)
Supplement: S2 Table — (DOCX) [file pone.0268771.s002.docx]

| Supplementary Table 2. Cox Proportional Hazards Model of 1-Year Post-Transplant Mortality: Era 1 | | | |
| --- | --- | --- | --- |
| Variable | Hazard Ratio | 95% CI | p-value |
| ECMO at transplant | 3.78 | 1.88 - 7.61 | < 0.001 |
| Age, per 1 y | 1.02 | 1.01 - 1.03 | < 0.001 |
| Female | 1.58 | 1.23 - 2.03 | < 0.001 |
| Body mass index, per 1 kg/m2 | 1.03 | 1.01 - 1.06 | 0.01 |
| Ventilator use at transplant | 2.53 | 1.16 - 5.55 | 0.02 |
| Prior cardiac surgery | 1.34 | 1.06 - 1.69 | 0.02 |
| Dialysis | 2.78 | 1.57 - 4.93 | < 0.001 |
| Cerebrovascular disease | 0.40 | 0.21 - 0.79 | < 0.01 |
| Functional status, per 1 u* | 1.02 | 0.97 - 1.08 | 0.42 |
| Serum creatinine, per 1 mg/dL | 1.34 | 1.16 - 1.56 | < 0.001 |
| Serum total bilirubin, per 1 mg/dL | 1.06 | 1.04 - 1.09 | < 0.001 |
| Systolic PA pressure, per 1 mmHg | 1.01 | 1.00 - 1.02 | 0.04 |
| Cardiac output, per 1 L/min | 1.08 | 0.98 - 1.19 | 0.14 |
| *ECMO = Extracorporeal Mechanical Oxygenation; PA = Pulmonary Artery* | | | |
| **Karnofsky functional status; lower numbers denote sicker patients* | | | |
