# Supplementary material for: Outcomes of extracorporeal membrane oxygenation following the 2018 adult heart allocation policy
Source: PLoS One. 2022 May 20;17(5):e0268771. doi: 10.1371/journal.pone.0268771 (PMC9122227; doi:10.1371/journal.pone.0268771)
Supplement: S3 Table — (DOCX) [file pone.0268771.s003.docx]

| Supplementary Table 3. Cox Proportional Hazards Model of 1-Year Post-Transplant Mortality: Era 2 | | | |
| --- | --- | --- | --- |
| Variable | Hazard Ratio | 95% CI | p-value |
| ECMO at transplant | 1.03 | 0.60 – 1.76 | 0.91 |
| Age, per 1 y | 1.02 | 1.00 - 1.03 | < 0.001 |
| Female | 1.23 | 0.94 – 1.60 | 0.13 |
| Body mass index, per 1 kg/m2 | 1.06 | 1.03 - 1.08 | < 0.001 |
| Ventilator use at transplant | 2.18 | 1.15 - 4.11 | 0.02 |
| Prior cardiac surgery | 1.66 | 1.30 – 2.11 | < 0.001 |
| Dialysis | 1.97 | 1.06 – 3.66 | 0.03 |
| Cerebrovascular disease | 1.08 | 0.69 – 1.69 | 0.73 |
| Functional status, per 1 u* | 0.98 | 0.92 – 1.03 | 0.43 |
| Serum creatinine, per 1 mg/dL | 1.13 | 1.00 - 1.29 | 0.06 |
| Serum total bilirubin, per 1 mg/dL | 1.04 | 1.01 - 1.07 | < 0.01 |
| Systolic PA pressure, per 1 mmHg | 1.00 | 0.99 - 1.01 | 0.48 |
| Cardiac output, per 1 L/min | 1.01 | 0.92 - 1.11 | 0.82 |
| *ECMO = Extracorporeal Mechanical Oxygenation; PA = Pulmonary Artery* | | | |
| **Karnofsky functional status; lower numbers denote sicker patients* | | | |
