# Supplementary material for: Outcomes of extracorporeal membrane oxygenation following the 2018 adult heart allocation policy
Source: PLoS One. 2022 May 20;17(5):e0268771. doi: 10.1371/journal.pone.0268771 (PMC9122227; doi:10.1371/journal.pone.0268771)
Supplement: S4 Table — (DOCX) [file pone.0268771.s004.docx]

| Supplementary Table 4. Cox Proportional Hazards Model of 1-Year Post-Transplant Mortality: ECMO Patients | | | |
| --- | --- | --- | --- |
| Variable | Hazard Ratio | 95% CI | p-value |
| Era 1 | Reference | Reference | Reference |
| Era 2 | 0.32 | 0.14 – 0.71 | < 0.01 |
| Age, per 1 y | 1.04 | 1.00 - 1.07 | 0.02 |
| Female | 1.24 | 0.48 – 3.22 | 0.65 |
| Body mass index, per 1 kg/m2 | 1.13 | 1.03 - 1.23 | < 0.01 |
| Ventilator use at transplant | 2.55 | 1.14 – 5.72 | 0.02 |
| Prior cardiac surgery | 2.33 | 1.08 – 5.00 | 0.03 |
| Dialysis | 1.36 | 0.49 – 3.76 | 0.55 |
| Cerebrovascular disease | 0.64 | 0.15 – 2.83 | 0.56 |
| Functional status, per 1 u* | 1.09 | 0.90 – 1.33 | 0.37 |
| Serum creatinine, per 1 mg/dL | 1.47 | 0.86 – 2.49 | 0.16 |
| Serum total bilirubin, per 1 mg/dL | 1.08 | 1.01 - 1.16 | 0.01 |
| Systolic PA pressure, per 1 mmHg | 1.01 | 0.99 - 1.04 | 0.44 |
| Cardiac output, per 1 L/min | 1.12 | 0.86 - 1.45 | 0.40 |
| *ECMO = Extracorporeal Mechanical Oxygenation; PA = Pulmonary Artery* | | | |
| **Karnofsky functional status; lower numbers denote sicker patients* | | | |
